# Surfactant-activated lipase hybrid nanoflowers with enhanced enzymatic performance

**DOI:** 10.1038/srep27928

**Published:** 2016-06-14

**Authors:** Jiandong Cui, Yamin Zhao, Ronglin Liu, Cheng Zhong, Shiru Jia

**Affiliations:** 1 Research Center for Fermentation Engineering of Hebei, College of Bioscience and Bioengineering, Hebei University of Science and Technology, 26 Yuxiang Street, Shijiazhang 050000, P R China; 2 Key Laboratory of Industrial Fermentation Microbiology, Ministry of Education, Tianjin University of Science and Technology, No 29, 13th, Avenue, Tianjin Economic and Technological Development Area (TEDA), Tianjin 300457, P R China

## Abstract

Increasing numbers of materials have been extensively used as platforms for enzyme immobilization to improve catalytic performance. However, activity of the most of the enzymes was declined after immobilization. Here, we develop a surfactant-activated lipase-inorganic flowerlike hybrid nanomaterials with rational design based on interfacial activation and self-assembly. The resulting surfactant-activated lipase-inorganic hybird nanoflower (activated hNF-lipase) exhibited 460% and 200% higher activity than native lipase and conventional lipase-inorganic hybird nanoflower (hNF-lipase). Furthermore, the activated hNF-lipase displayed good reusability due to its monodispersity and mechanical properties, and had excellent long-time stability. The superior catalytic performances were attributed to both the conformational modulation of surfactants and hierarchical structure of nanoflowers, which not only anchored lipases in an active form, but also decreased the enzyme-support negative interaction and mass-transfer limitations. This new biocatalytic system is promising to find widespread use in applications related to biomedicine, biosensor, and biodiesel.

Enzymes as the potent biocatalysts have been used in biotechnology and pharmaceutical processes, and chemical industry for many years because enzymes are highly effective and versatile biological catalysts that exhibit high chemo-, regio- and enantio-selectivity at ambient temperatures^1^,^2^,^3^. However, most free enzymes display low stability and difficulties in recovery and reuse, hindering to their industrial use^4^,^5^,^6^. Immobilization of enzymes is a promising technology that can overcome these limitations. Generally, immobilized enzymes show improved stability, making them efficient, reusable and economical. The main reasons for stabilization of immobilized enzymes can be found in the prevention of subunit dissociation via multisubunit immobilization^7^, prevention of aggregation^8^, autolysis or proteolysis by proteases^9^, rigidification of the enzyme structure via multipoint covalent attachment^10^. In addition, immobilization may also generate a favorable microenvironment^11^. However, activity of the most of the enzymes was reduced after immobilization due to the partially blocking of enzyme active sites during the immobilization process, the enhanced mass-transfer limitations between the enzyme and substrate, and conformational changes in the enzyme, which are harmful to their catalytic performances^12^,^13^,^14^. Therefore, development of a simple and efficient approach for immobilized enzymes with enhanced catalytic activity and stability is highly desirable. Recently, an elegant approach in enzyme immobilization was developed by Zare and coworkers^12^. They created organic-inorganic hybrid nanoflowers consisting of protein and metal ions, which resulted in much higher enzyme activity and stability than free enzymes and conventional immobilized enzymes. As the following work, Lin *et al*. used Cu_3_(PO_4_)_2_·3H_2_O-horserdish peroxidise hybrid nanoflowers as sensors for detection of hydrogen peroxide and phenol^13^, while Wang *et al*. prepared CaHPO_4_-α-amylase hybrid nanoflowers for a nanobiocatalytic system using the same synthetic route^14^. In addition, a multi-enzyme (glucose oxidase and horseradish peroxidase) co-embedded organic–inorganic hybrid nanoflowers for a colorimetric sensor was also prepared by Sun *et al*.^15^. More recently, Yu *et al*. synthesized organic–inorganic nanoflowers for crude soybean peroxidase purification^16^. Somturk *et al*. utilized Cu_3_(PO_4_)_2_·3H_2_O-horserdish peroxidise hybrid nanoflowers as a sensor for the detection of dopamine^17^. Batule *et al*. developed an ultrafast sonochemical method to synthesize Cu_3_(PO_4_)_2_·3H_2_O-laccase hybird nanoflowers by sonication treatment^18^. Despite these reports, the underlying principles of nanoflower formation and increased catalytic activity and stability still remain elusive, and further studies are needed to explore new synthesis strategies, understand the interaction between the inorganic phase and enzyme and to elucidate the mechanism of enhancement of enzyme activity and stability.

Lipases are versatile biocatalysts that can catalyze various types of reactions, such as hydrolysis, esterification, inter-esterification and aminolysis under mild conditions, therefore, lipase was widely used in food, pharmaceutical, and chemical industry^19^. However, free lipase exhibits poor stability, the difficulties of recovery and recycle during bioconversion, which presents a significant barrier to lipase application^20^,^21^. Therefore, many attempts have been made to improving lipase activity and stability, such as genetic engineering, protein engineering, and chemical modification^22^,^23^,^24^. Unfortunately, these techniques are time-consuming, expensive, and tedious^25^. In contrast, enzyme immobilization provides the extra advantage of increasing catalytic activity and stability relative to that of free lipases^20^,^21^. Generally, most lipases are “inactive” closed form in aqueous media due to a hydrophobic amino acids chain that covers their active site, called lid, which confers the open-closed form of enzyme. Hence, to fully exploit the advantages of immobilized lipases, it is essential to anchor lipases in an active form while they are immobilized. In general, in presence of interface polar–non-polar, the lid of lipase is dislocated and the enzyme shows its opened and active form, which called interfacial activation behavior^26^,^27^ Moreover, the interfacial activation can induce a dramatic increase in catalytic activity of lipases^28^,^29^. This effect gave us the vital spark to anchor lipase with active form in hybird nanoflowers by intergrating interfacial activation and self-assembly. Therefore, in this study, we develop a novel immobilization strategy based on interfacial activation and hybird nanoflowers. The successful preparation of surfactant-activated hybrid nanoflower was investigated for the immobilization of lipase from bovine pancreatic. As illustrated in Fig. 1, lipases were first activated by surfactant based on interfacial activation mechanism. After that, enzyme-inorganic hybrid nanoflowers using Cu_3_(PO_4_)_2_ as the inorganic component and the activated lipases as the organic component (activated hNF-lipase) were synthesized by self-assembly. In other words, surfactants acts as an activator to open active form of lipase, subsequently, the open active form is anchored in hybird nanoflowers. For comparison, enzyme-inorganic hybrid nanoflowers using Cu_3_(PO_4_)_2_ as the inorganic component and lipases without activation as the organic component (hNF-lipase) were also prepared. Compared to free lipase and hNF-lipase, the activated hNF-lipase exhibited excellent catalytic activity and stability. The superiority of the activated hNF-lipase could be due to the following: (1) Surfactants induce the open conformation of lipase, which makes the active site accessible and, following immobilization, could fix the activated hNF-lipase in the “open conformation”^26^. (2) Proper interfacial activation could enhance protein rigidity and provide a more effective conformational stabilization of lipase in the activated hNF-lipase^28^. (3) Nanoflower-like structure of lipase with large surface area and extensive confinement could decrease mass-transfer limitations, which result in higher accessibility of the substrate to the active sites of the enzyme^13^,^14^. (4) Surfactants could prevent the formation of large lipases aggregates in nanoflowers^26^, which is helpful to generate uniform architectures with good monodispersity.

## Results

### Synthesis and characterization of activated hNF-lipase

**Figure 1:**
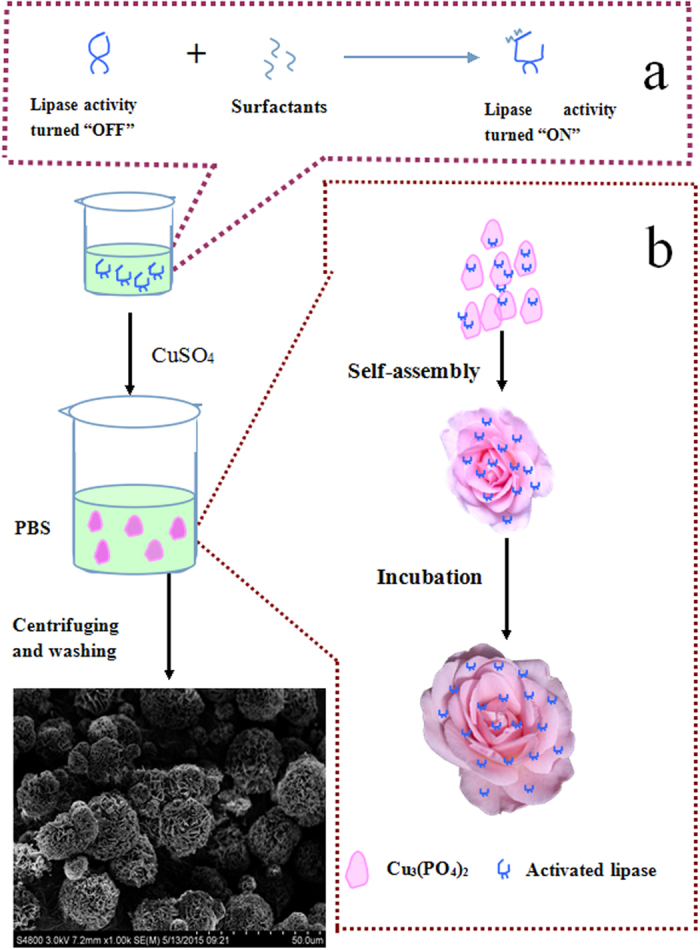
Schematic illustration of the synthesis strategy. (**a**) Lipases were activated by surfactants, (**b**) the synthesis of the activated hNF-lipase.

The synthesis of activated hNF-lipase involved two main steps (Fig. 1). First, lipases were activated with surfactants based on interfacial activation mechanism. As shown in Fig. 1a, in the absence of surfactants in an aqueous solution, most lipases are “inactive” because their active centers are covered by a polypeptide chain called lid. However, with the involvement of surfactants, lipases show opened and active form^26^. Second, aqueous CuSO_4_ solution was added to phosphate buffer (0.1 M, pH 7.5) solution containing activated lipases. After incubation at 4 °C for 3 d, activated hybrid nanoflowers were obtained (Fig. 1b). Herein, the mechanism of forming nanoflowers could comprise three steps: (1) nucleation and formation of primary crystals. (2) complex formation of lipase molecules with Cu^2+^ through coordination of amide groups in the lipase backbone, and (3) complete formation of nanoflowers^12^,^16^. To optimize the synthetic condition, the effect of lipase concentration on the formation of hybrid nanoflowers was investigated. As showed in Figure S1, amorphous bulky crystal-like structures, but no nanoflowers, were observed at lower lipase concentration (0.005 mg/mL and 0.01 mg/mL) (Figure S1a and S1b). However, the flower-like nanostructures began to appear with increasing lipase concentration (0.015 mg/mL) (Figure S1c). The uniform nanoflowers emerged when the concentration of lipase was 0.02 mg/mL (Figure S1d) and 0.025 mg/mL (Figure S1e). Upon the increase in the lipase concentration to 0.1 mg/mL and 0.5 mg/mL, flowerlike spherical structure (Figure S1f,g) was observed instead of nanoflowers. A further increase in the lipase concentration resulted in thicker nanosheets aggregates (Figure S1h). Similarly, nanoflowers were formed only if the concentration of CuSO_4_ was greater than 1.2 mM (Figure S2b–d). Otherwise, nanosheets were generated at lower CuSO_4_ concentration (Figure S2a). The results indicated that lipase and CuSO_4_ concentration added in the reaction synthesis responded to the morphology and size of the hybrid nanoflowers, which would affect the activity and stability of the immobilized enzyme.

**Figure 2:**
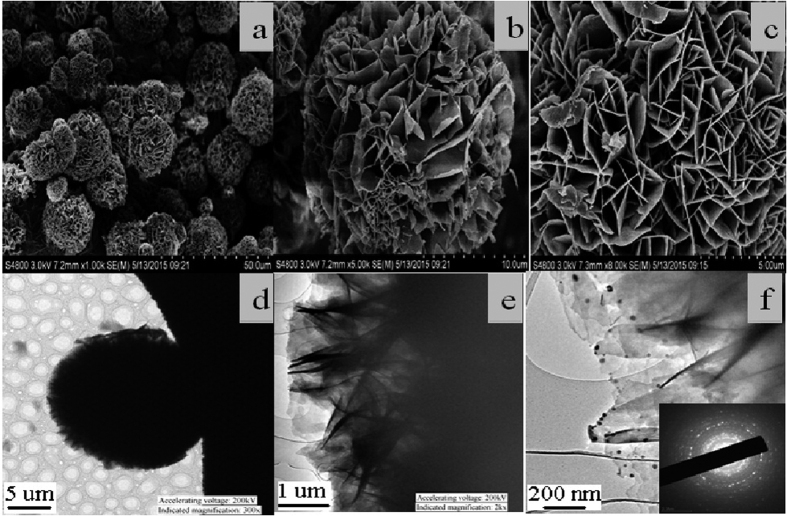
SEM images of (**a**–**c**) the activated hNF-lipase; TEM images of (**b**,**d**,**f**) the activated hNF-lipase.

**Figure 3:**
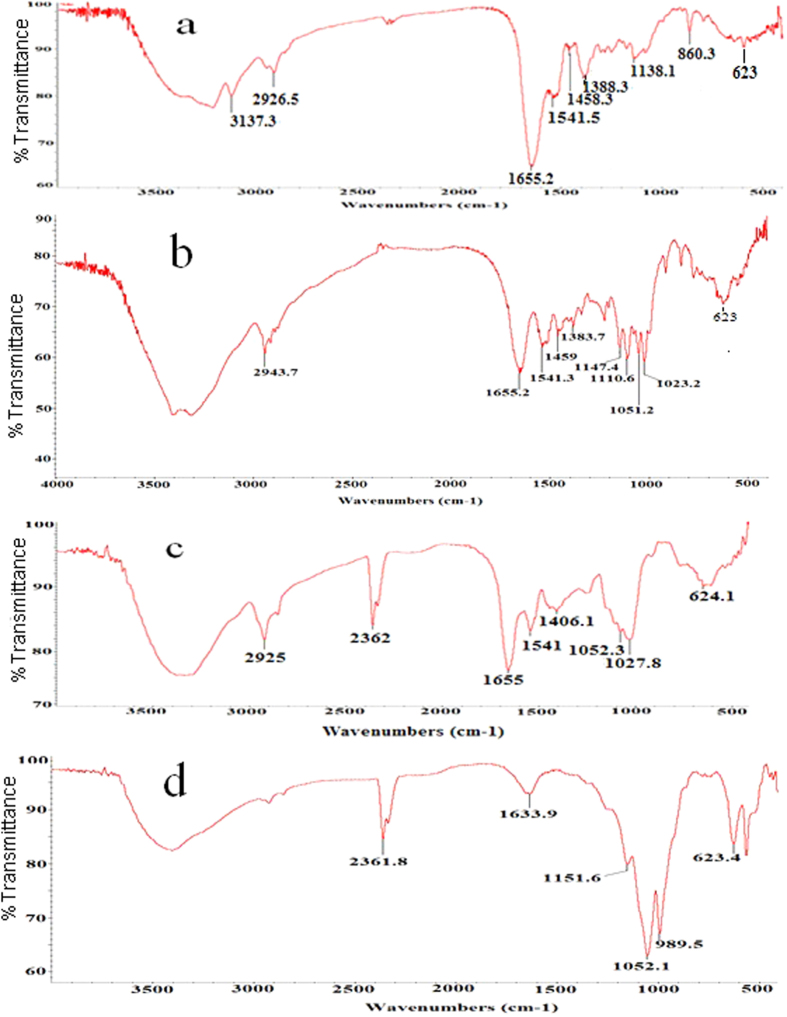
FT-IR spectra analysis. (**a**) lipase, (**b**) hNF-lipase, (**c**) activated hNF-lipase, (**d**) Cu_3_(PO_4_)_2_.

**Figure 4:**
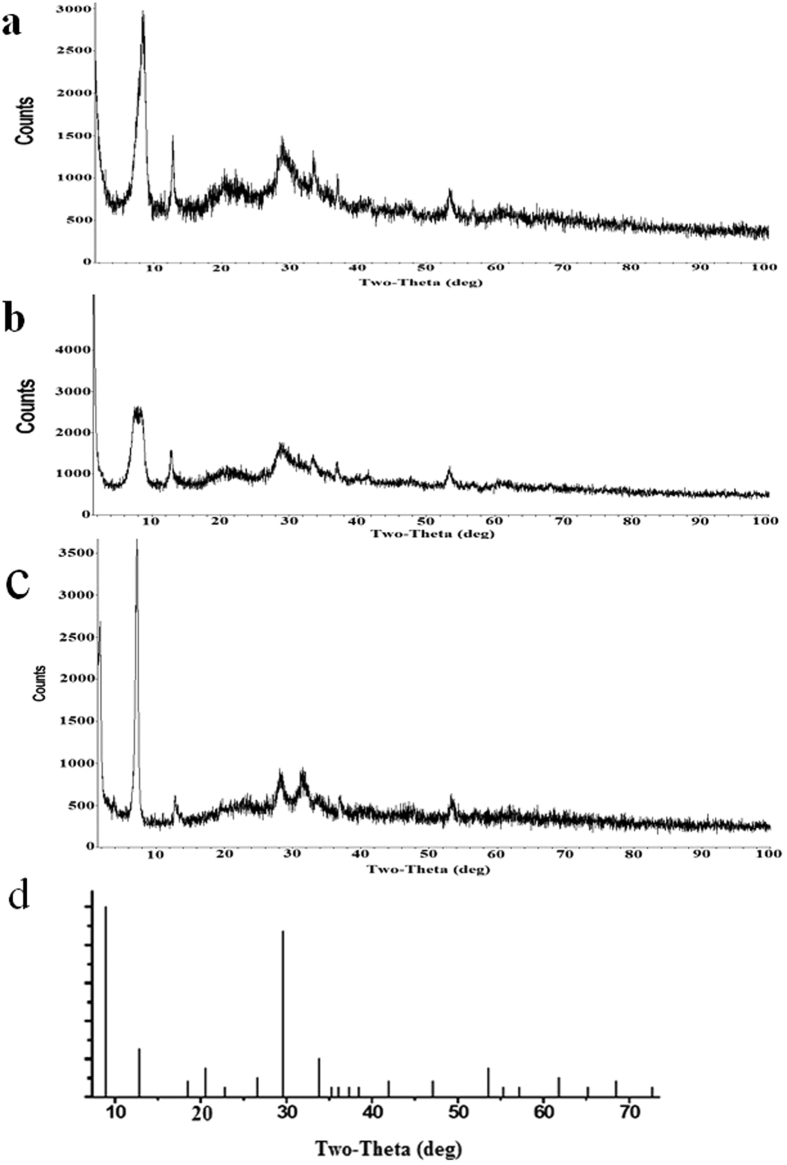
XRD patterns of the activated hNF-lipases formed with 0.025 mg/mL lipase. (**a**) hNF-lipase, (**b**) activated hNF-lipase, (**c**) particles of crystals obtained without lipase, (**d**) standard Cu_3_(PO_4_)_2_·3H_2_O (JPSCD 00-022-0548).

**Figure 5:**
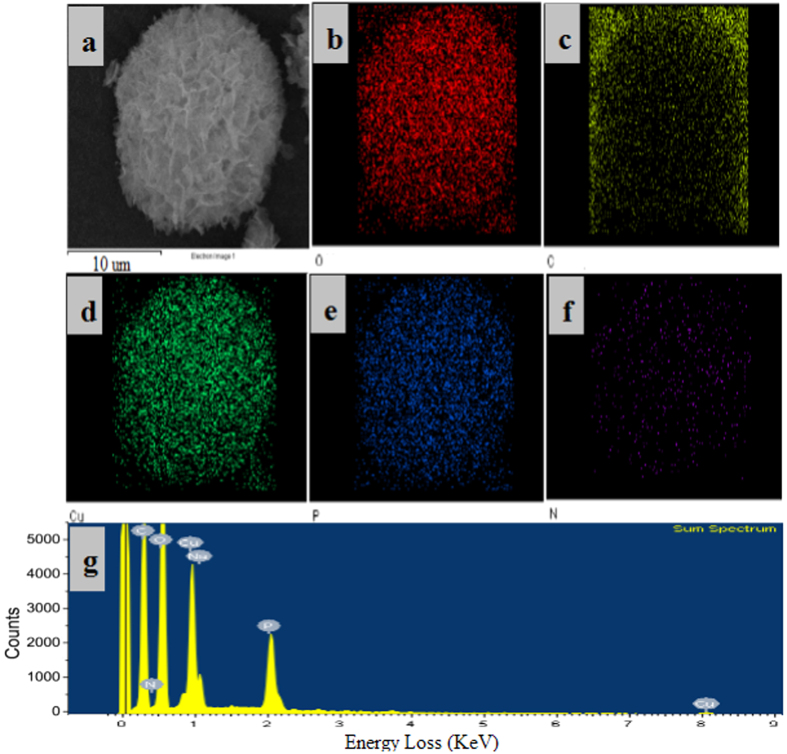
Element mapping of the activated hNF-lipase via EDS: (**a**) the sample; images (**b**–**f**) exhibit the element sensitive maps of carbon, oxygen, copper, phosphorus, and nitrogen; (**g**) EDS spectrum of complete element distribution.

**Figure 6:**
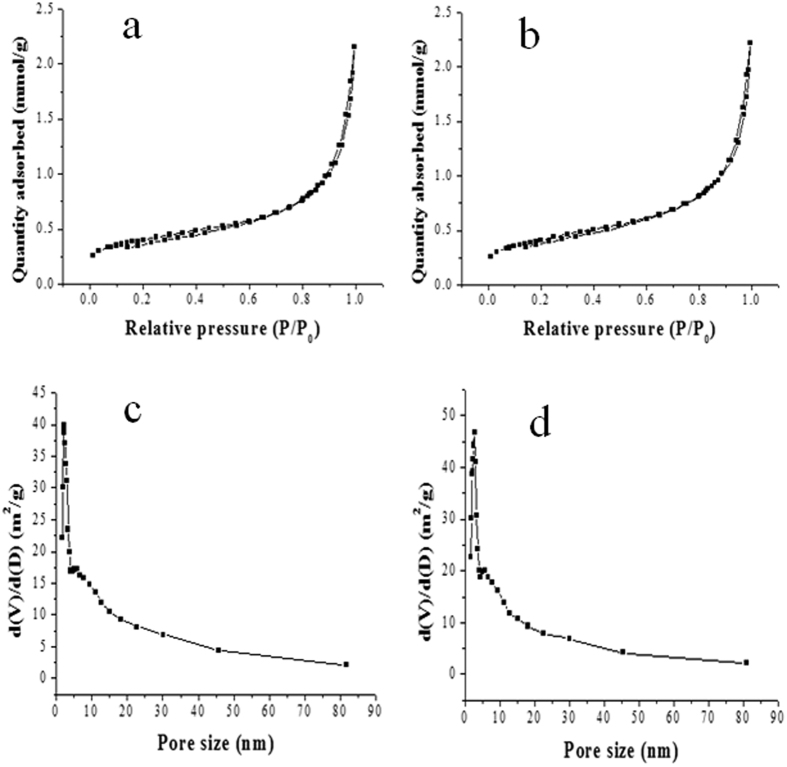
N_2_ adsorption-desorption isotherms and pore size distribution curves of the hNF-lipase and activated hNF-lipase samples. (**a**,**c**) hNF-lipase, (**b**,**d**) activated hNF-lipase.

Figure 2 displayed the SEM and TEM images of the activated hNF-lipase. In the low-resolution SEM images, most of the hybrid nanoflowers were uniform architectures with good monodispersity (Fig. 2a). High-resolution SEM image (Fig. 2b,c) displayed that the hybrid nanoflowers had hierarchical peony-like flower morphology with an average size of ~20 μm, which was assembled from hundreds of nanoplates. TEM image of the single nanoflower was showed in Fig. 2d,e, in which we can clearly find that nanoflowers are built up by interlaced nanoplates. Selective area electron diffraction analysis revealed that the two axes in the nanoflowers were aligned in the (012) and (017) directions (Fig. 2f). In addition, the chemical structures of hNF-lipase and activated hNF-lipase were also monitored by FTIR. Two regions (1700 cm^−1^ to 1600 cm^−1^ and 1550 cm^−1^ to 1500 cm^−1^) were found in the Fig. 3a,c, which was unique to the protein secondary structure. Such regions were designated as amides I and II, respectively^30^. In Fig. 3a, typical absorption peaks of native lipase occurred at 1655 and 1541 cm^−1^ for CONH and 2800–3000 cm^−1^ for CH_2_ and CH_3_. The same absorption peaks at 1655 and 1541 cm^−1^ and 2800–3000 cm^−1^ were also observed in spectrum of the hNF-lipase and the activated hNF-lipase (Fig. 3b,c). The results indicated that lipases were encapsulated in nanoflowers. Furthermore, characteristic absorption (Fig. 3b–d) at 1052 cm^−1^ and 623 cm^−1^ may be attributed to P−O vibrations; these signal indicated the presence of phosphate groups^16^. The XRD patterns in Fig. 4 revealed the crystallographic structures of the nanoflowers and Cu_3_(PO_4_)_2_·3H_2_O. The results exhibited the positions and relative intensities of all diffraction peaks in Fig. 4a–c matched well with those obtained from the JCPDS card (00−022−0548) (Fig. 4d), which indicated that the hybrid nanoflowers were well crystallized and had high crystallinity after incorporating lipase. EDS experiment revealed that five typical elements including Cu, P, C, O, and N (Fig. 5(b–f) were observed in the activated hNF-lipase and attributed to Cu_3_(PO_4_)_2_/lipase nanocomposites. The complete element distribution was shown in the EDS spectrum in Fig. 5g. The similar results were also observed in the hNF-lipase (Figure 3S), indicating that the nanoflowers were composed of Cu_3_(PO_4_)_2_ crystals dispersed into the organic lipase component. Besides, we also determined the mass losses of samples of the activated hNF-lipase by TGA. It is revealed that the weight percentage of organic component (i.e., lipase) of the nanoflowers was 15.26% (Figure S4), signifying an effective hybridization of Cu_3_(PO_4_)_2_ with lipase. Taken together, we show that enzyme-inorganic hybrid flowers using Cu_3_(PO_4_)_2_ as the inorganic component and the activated lipases with surfactants as the organic component were successfully synthesized by self-assembly. In addition, the pore size distribution in the hNF-lipase and the activated hNF-lipase was also determined by nitrogen gas adsorption/desorption analysis. All hybrid nanoflowers displayed a type IV isotherm with H_1_ hysteresis, which is typical of mesoporous structures (Fig. 6a,b)^31^,^32^. The hNF-lipase and activated hNF-lipase had the multiple level pore size distribution (a sharp band centered at pore size of 2.16 nm and 2.7 nm and a broad band between 3 and 80 nm, respectively) (Fig. 6c,d), manifesting their porous and hierarchical structures. Moreover, The BET specific surface area for hNF-lipase and activated hNF-lipase was around 31.04 m^2^/g and 32.42 m^2^/g, respectively (Table 1). These porous and hierarchical structures are critical for ensuring high catalytic efficiency, due to higher accessibility of the substrate to the active sites of the enzyme.

### Activity of the activated hNF-lipase

**Figure 7:**
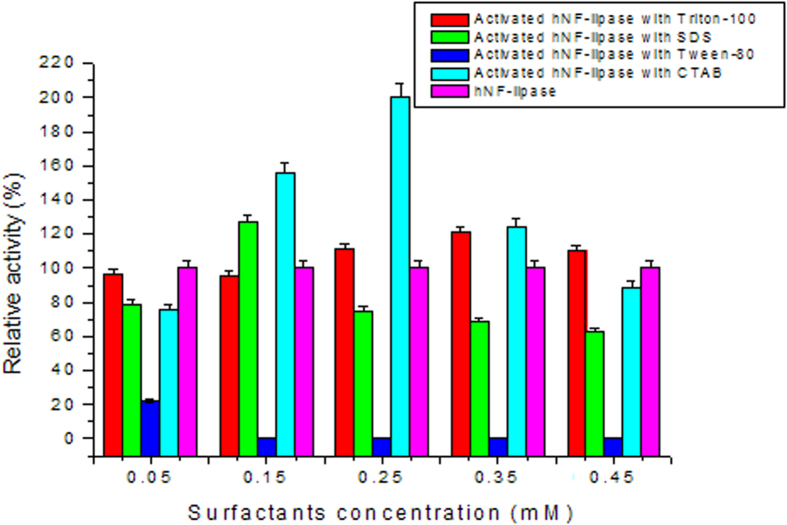
Effect of different surfactants concentration on the the activated hNF-lipases activity.

To improve the catalytic performance of hNF-lipase, a new strategy of integrating interfacial activation and hybrid nanoflowers was developed. Lipases were first activated by surfactants based on interfacial activation mechanism. The activated lipases were sequentially used for hNF-lipase preparation. The relative activity of the activated hNF-lipase was calculated by taking the enzyme-catalyzed activity of the hNF-lipase as 100%. The results were shown in Fig. 7. Compared with the hNF-lipase, the activated hNF-lipase with CTAB exhibited a significant hyperactivation in lipase hydrolytic activity at 0.25 mM concentration. The relative activity was increased 200%. Furthermore, the enzymatic activity of lipase in the activated hNF-lipase was approximately 460% higher than free lipase in solution. The results indicated that CTAB had a significant hyperactivation in activity of bovine pancreatic lipase. However, the addition of Triton X-100 had a minor effect on the activity of the hNF-lipase compared with that of CTAB, suggesting that Triton X-100 could not significantly induce effective activation of the lipase. Furthermore, complete inhibition of catalytic activity of lipase was observed in the presence of Tween-80. These results indicated that different surfactants exhibited positive or negative effect in the activity of lipases. Therefore, CTAB was selected as the optimal amphiphile for the activation of bovine pancreatic lipase and the optimum concentration was 0.25 mM.

The hyperactivation of the activated hNF-lipase with CTAB could be due to the following: on the one hand, CTAB could induce the open conformation of lipases, which makes the active site accessible and, following immobilization, could fix the activated hNF-lipase in the “open conformation”. To verify the conformational change of lipase, FTIR experiments were performed to analyze the secondary structure variation with the conditions described in Materials and Methods section. FTIR technique renders feasible the study of a protein’s secondary structure since proteins absorb infrared wavelengths due to the peptide bond vibrations^33^,^34^. Furthermore, the amide I region at approximately 1600–1700 cm^−1^ is mostly used in protein secondary structure determination due to its sensitivity in conformational changes and the significantly higher signal intensity than in other amide bands^35^. The results revealed that the predominant absorbance spectra in amide I band were α-helix: 1650–1658 cm^−1^, β-sheet: 1620–1640 cm^−1^, β-turn: 1670–1695 cm^−1^, and random coli: 1640–1650 cm^−1^, respectively. The position and number of the secondary structural components for free lipase, hNF-lipase, and activated hNF-lipase were shown in Figure S5. A Gaussian-sum function was used to fit the overlapped bands, measuring peak position and areas. The best fit was used to estimate the percentage contribution of each band to the spectrum of amide I. The percentage of secondary structures for lipase estimated from the FTIR was shown in Table 2. The lipase exhibited some variations in the contents of α-helix, β-turn, β-sheet and random coil. Compared with free lipase and hNF-lipase, the activated lipase in hybird nanoflows showed a decrease in α-helix and β-turn content and an increase in β-sheet and random coil content. It has been confirmed that the active site of lipase is covered by a lid, consisting of **α**-helix^36^. The lid was found to be a structural and functional determinant of lipase activity. The decrease in **α**-helix contents of lipase affects the lipase active site by stimulating higher tendency for the “open” conformation, which allows easier access to the substrate. Furthermore, studies have demonstrated that the lipase activity increased with the decrease of **α**-helix content^37^. In conclusion, important conformational changes of lipase occur due to interfacial activation behavior, the lid of lipase is dislocated and the enzyme shows its opened and active form.

On the other hand, CTAB may also prevent the formation of large lipase aggregates. As a result, lipases were uniformly dispersed in nanoflowers. This reason was confirmed by the magnified TEM. The several large lipase aggregates were observed in TEM image of the hNF-lipase (Figure S6a), whereas the large lipase aggregates were not found in TEM image of the activated hNF-lipase (Figure S6b). Furthermore, SEM image revealed that the hNF-lipase exhibited the broad distribution in particles size (Figure S6c). However, the sizes of the activated hNF-lipase particles were relative uniform (Figure S6d). This difference was further confirmed by fluorescence micrograph using FITC labeled lipases since the labeled lipases showed a typical green fluorescence image (Figure S6e and S6f). The broad distribution in the hNF-lipase particle size could be attributed to the formation of lipases aggregates.

Based on the enzyme amounts in solution before and after the immobilization measured with the Bradford method, immobilization efficiency (encapsulation yields) of lipases in hybird nanoflowers formed from 0.025, 0.1, 0.25, 0.5, and 1 mg/mL lipases in solution was determined, respectively. The results were shown in Table S1, the encapsulation yield of lipases in the nanoflowers decreased from 87% to 35% with lipase concentration changing 0.025 to 1 mg/mL. The results were in accordance with the previous reports^14^,^16^. On the basis of the above results, the hybrid nanoflowers with 0.025 mg/mL lipase was chosen as the best for further evaluation and applications considering its good morphology and high encapsulation yield.

Besides, the kinetic parameters of free lipase, hNF-lipase, and activated hNF-lipase were determined by calculating the initial rates at various substrate concentrations. Table 3 shows the *V*_*max*_, *K*_*m*_, and *V*_*max*_/*K*_*m*_ values for the three forms of lipase. It was found that activated hNF-lipase undertook the lowest *K*_*m*_ value, confirming that they have a higher affinity of substrate toward lipase molecules compared to free lipase and the hNF-lipase. Moreover, the *V*_max_ and *V*_max_/*K*_*m*_ of activated hNF-lipase were significantly increased. We attribute this increased catalytic efficiency (*V*_max_/*K*_*m*_) to the following effects: (1) high surface area and porosity of hybird nanoflowers, (2) the open conformation of lipase in the activated hNF-lipase, (3) synergistic effect of nanoscale-entrapped lipase.

In addition, the effect of reaction temperature on the activity of free lipase, hNF-lipase, and activated hNF-lipase was also examined. As showed in Figure S7, the optimum temperature of free lipase and hNF-lipase was similar, and all demonstrated their highest activity at 50 °C. However, the optimum temperature of activated hNF-lipase determined to be 40 °C. The decreased optimum reaction temperature of activated hNF-lipase could be due to the fact that surfactants induce the open conformation of lipase, which makes the active site accessible and, following immobilization, could fix the activated lipase in the “open conformation”, which leading to lower activation energy requirement at the surface of nanoflowers particles. The results suggested that the activated hNF-lipase can be used at lower temperatures, which is more energy-efficient and environmentally friendly.

### Stability of the activated hNF-lipase

**Figure 8:**
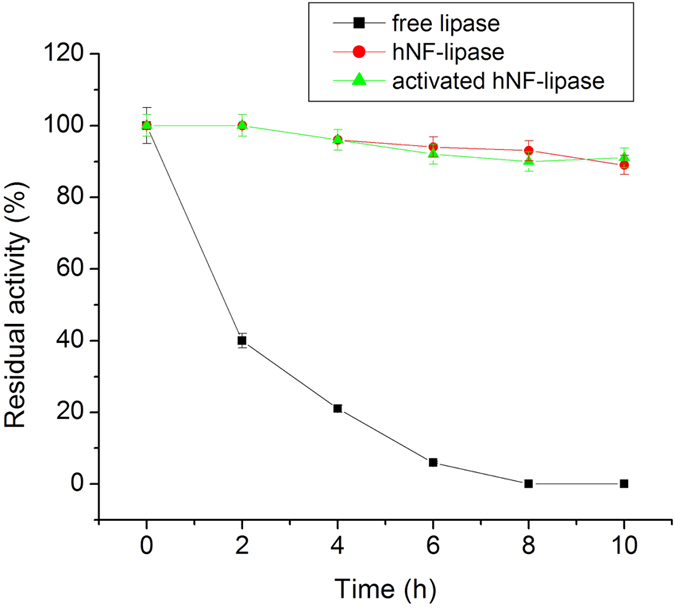
Thermal stability of free lipase, hNF-lipase, and activated hNF-lipase at 60 °C.

**Figure 9:**
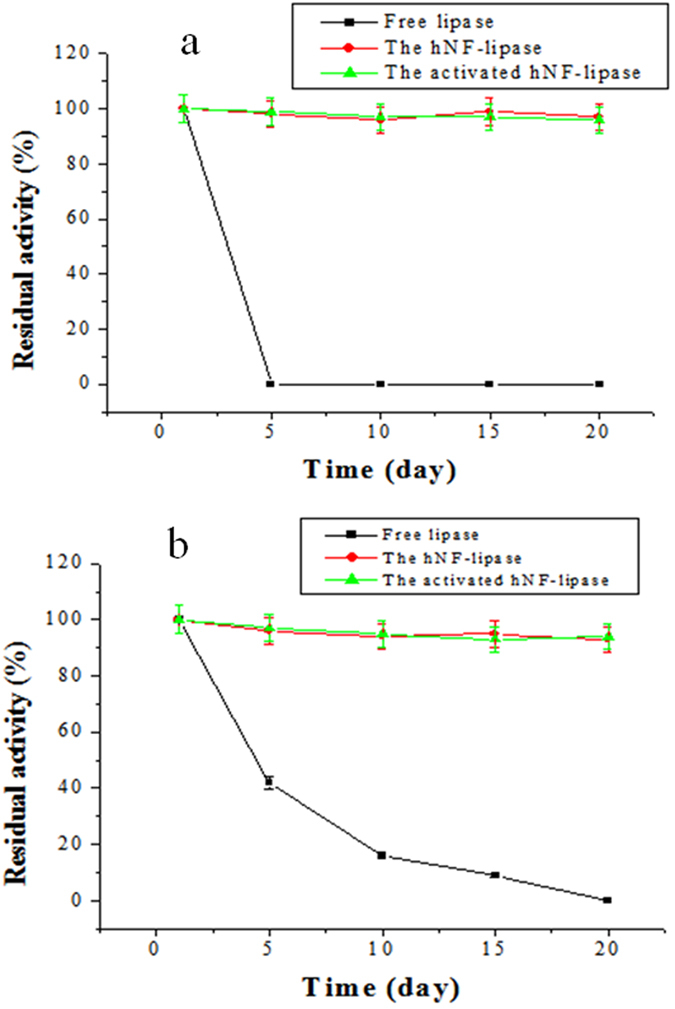
Mechanical stability (**a**) and storage stability (**b**) of free lipase, hNF-lipase, and activated hNF-lipase.

Stabilization of the enzyme is compulsory for synthetic application of the immobilized enzymes. Therefore, the stability of free lipase, hNF-lipase, and activated hNF-lipase against heating was compared. The results (Fig. 8) revealed that hNF-lipase and activated hNF-lipase showed more stable performance than free lipase at 60 °C. For example, hNF-lipase and activated hNF-lipase still retained 92% and 93% of the original activity even at 60 °C for 10 h, respectively, whereas free lipase almost lost activity at the same conditions. A similar phenomenon was also observed while assessing pH tolerance of free lipase and nanoflowers against extreme pH. As shown in Figure S8, the hNF-lipase and activated hNF-lipase were more stable than the free lipase between pH 4 and 12, the pH stability range was broadened from 8–10 to 4–12. The higher pH and temperature resistance of hybird nanoflowers are assumed to be due to the the enzyme molecules confined in the nanoporous structures that increases the rigidity of enzyme. In addition, to test the stability of free lipase, hNF-lipase, and activated hNF-lipase against mechanical damage and leaching, free lipase and immobilized lipase were incubated in sodium phosphate buffer solution (pH 7.5) at 25 °C and shaken at 200 rpm for a certain time. The results were shown in Fig. 9a. Free lipase activity decreased considerably and no activity was determined by 5 days. However, hNF-lipase, and activated hNF-lipase retained 96% and 95% of its initial activity after 20 days of shaking, respectively. No significant enzyme leaching was observed by SDS-PAGE during shaking (Figure S9), and lipases were still encapsulated in the nanoflowers after 30 days of shaking as evidenced by LCSM image (Figure S10). Furthermore, no pronounced changes in sizes and morphologies were found during the whole shaking period, indicating that neither collapse nor aggregation occurred and the porous and flowerlike structures were well maintained (Figure S11), demonstrating that the nanoflowers process very good mechanical stability. Similar results were observed for the storage stability. The hNF-lipase and activated hNF-lipase retained about 94% and 95% of its initial activity at 25 °C for 20 days storage, respectively. However, free lipase lost most of its activity during the same storage period (Fig. 9b). The good mechanical stability and storage stability of the hybrid nanoflowers were ascribed to the appropriate stability of copper phosphate and the entrapment of the lipase molecules in the nanoflowers, which prevented their escape.

**Figure 10:**
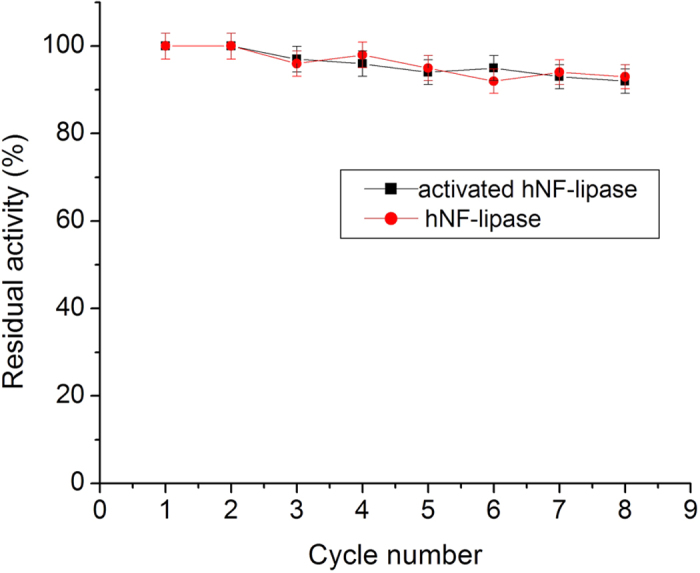
Stability of hNF-lipase and activated hNF-lipase during repeat cycles.

For any industrial application, the reusability of immobilized enzymes is a key factor for its cost-effective use^38^. Therefore, the reusability of the hybrid nanoflowers was tested. As shown in Fig. 10, although the activities of hNF-lipase and activated hNF-lipase slightly decayed with increasing number of recycles, they still retained more than 90% of its initial activity until 8 cycles. Furthermore, it is worth pointing out that the nanoflowers did not occur obvious morphological change (Figure S12a and S12b), and lipase molecules were still entrapped in nanoflowers (Figure S12c and S12d) after eight rounds of successive catalytic reaction, indicating that the nanoflowers were excellent reusability and reproducibility.

## Discussion

Recently, as a newly developed class of immobilization enzymes, hybrid nanoflowers have attracted attention due to their simple synthesis, high activity, and stability^36^. Here, we have first developed a new concept to impart new functions to biocatalysts by combining interfacial activation mechanism of lipase and enzyme-inorganic hybird nanoflower. The proof-of-concept design is demonstrated by embedding active form of lipase molecules into uniformly sized nanoflowers via self-assembly. Namely, in this stratage, the surfactant was first used as an activating agent to open active form of lipase, subsequently, the lipases with an active form were anchored in hierarchical nanoflowers via self-assembly. The resulting activated hNF-lipase offered a dramatically enhancement in activity and exhibited superior stability, with a higher retention of activity, eliminating enzyme aggregation and leaching, tolerance to high temperature, prolonged storage, and continuous recycles, which could be ascribed to the synergic effect generated from the the conformational modulation of surfactants and hierarchical structure of nanoflowers. The synergic effect not only anchored lipases in an active form, but also decreased the enzyme-support negative interaction and mass-transfer limitations. We anticipate that this proof of concept can be applied in several fields including biosensors, biomedicine, analytical devices, biofuel cells, and industrial biocatalysis.

## Methods

### Materials

Lipase (EC 3.1.1.3 Type II, from bovine pancreas), р-nitrophenol (рNP), and 4-Nitrophenyl acetate (4NPA) (99%) was purchased from International Aladdin Reagent Inc. (Shanghai, China), and stored at 4 °C. The activity is 15–30 U/mg. Copper sulfate pentahydrate (CuSO_4_·5H_2_O), hexadecyl trimethyl ammonium bromide (CTAB), Triton X-100, Tween-80, and sodium dodecyl sulfate (SDS) was obtained from Beijing Chemical Reagent Company (Beijing, China). Fluorescein isothiocyanate (FITC) was obtained from Sigma-Aldrich Inc. (St. Louis, MO, USA). Other reagents used were of analytical grade.

### Preparation of hNF-lipase and activated hNF-lipase

Hybrid nanoflowers were prepared as previously reported with some modifications^39^. For activated hNF-lipase, first, 0.02 mg/mL of lipase in a 0.1 M phosphate buffer solution (300 ml, pH 7.5) and appropriate amounts of surfactant were mixed and stirred at 4 °C for 30 min. The mixture solution was incubated at 4 °C for 24 h to achieve the activated lipase solution. Second, 2 ml of CuSO_4_ aqueous solution (120 mM) was added to the activated lipase solution (300 ml). The reaction was then allowed to proceed at 4 °C for 72 h. Blue precipitates were collected after centrifugation and dried under vacuum at room temperature. The hNF-lipase was prepared as the general procedure, but no adding surfactant into the enzyme solution. In addition, effects of lipase concentration (0.005, 0.01, 0.015, 0.02, 0.025, 0.1, 0.5, 5 mg/mL) and CuSO_4_ concentration (0.4, 1.2, 2, 3.2 mM) on the formation hybrid nanoflowers were investigated, respectively.

### Activity assay

The activities of free lipase and hybrid nanoflowers to catalyze the hydrolysis of 4NPA to рNP was measured following the procedures described by Pereira *et al*.^40^. The substrate solution contained 50 mM sodium phosphate buffer solution (pH 7.5), 0.5 mM 4NPA and 0.2% Triton X-100. Then a small amount of enzyme sample was added to substrate solution. The resultant reaction medium was incubated at 37 °C for 5 min. The рNP formation was monitored at 410 nm using a 2800H spectrophotometer (Unicoi Instrument Co., Ltd. Shanghai). One unit of lipase activity was defined as the amount of lipase releasing 1 μ mol of рNP from 4NPA per minute. The lipase immobilization efficiency was calculated using the following equation:


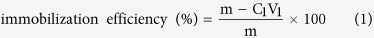


where m (mg) represents the mass of lipase initially added to the solution, C_1_ (mg/mL) represents the lipase concentration of the supernatant, and V_1_ (ml) represents the volume of the supernatant.

### Characterization

Scanning electron microscope (SEM) was taken by JEOL JSM6700. Transmission electron microscope (TEM) images were obtained on JEOL JEM2100. Confocal laser scanning microscopy (CLSM) was used to investigate the presence of luminescent-tagged lipases within the nanoflowers. Prior to observation, lipases were mixed with FITC solution (50 mg/mL, FITC in acetone) for 3 min. Modified FITC labeled lipases were then immobilized. CLSM observation was performed with a Leica TCS SP5 microscope (Leica Camera AG, Germany). The samples were excited at 390 nm and FITC fluorescence was detected between 460 and 480 nm. N_2_ adsorption isotherms were obtained on a Beckman coulter SA3100 analyzer at 77 K. Specific surface areas and pore diameter distribution were calculated using Brunauer-Emmett-Teller (BET) and Barrett–Joyner–Halenda (BJH) models, respectively, based on the adsorption isotherm. Fourier transform infrared (FTIR) spectra of free lipase and immobilized lipase were obtained using a NEXUS870 infrared spectrometer (Thermo Nicolet Corporation, Madison, WI) using the standard KBr disk method. FT-IR measurements were conducted in the region of 400–4000 cm^−1^. The secondary structure element concent was estimated based on the information of amide I region and the band assignment using Omnic software 8.0 (Thermo Nicolet corporation, USA) and Peakfit software 4.0 (SeaSolve Software Inc. USA) according to the method describled by Liu *et al*.^41^. The crystal structures of the nanoflowers were determined by X-ray powder diffraction (XRD) (D/Max-2500 diffractometer, Shimadzu, Japan). The elemental composition of the nanoflowers was analyzed by using energy-dispersive spectrometer (EDS) (S2 Ranger, Bruker, Germany). Thermal gravimetric analysis (TGA) measurements were performed on SDT Q600 (TA Instruments-Waters LLC, USA). The samples were filled into an alumia crucibel and heated in a continuous flow of nitrogen gas with a ramp rate of 10 °C/min from 25 up to 800 °C.

### Measurement of kinetic parameters

The kinetic parameters, *K*_m_ and *V*_max_, for free lipase, hNF-lipase, and activated hNF-lipase were calculated by the Lineweaver-Burk double-reciprocal plot method of Michaelis-Menten Equation between 0.08 and 0.12 mM 4NPA concentrations at a constant enzyme concentration (0.1 mg/mL). The enzymatic reaction was carried at pH 7.5 (50 mM phosphate buffer), 37 °C, and the change in absorbance was measured at 410 nm.

### The stability of free lipase, hNF-lipase, and activated hNF-lipase

The time courses of thermal inactivation of free lipase, hNF-lipase, and activated hNF-lipase were investigated by incubating them in 50 mM sodium phosphate buffer solution (pH 7.5) without substrate at 60 °C for 2–10 h, and the enzyme samples were taken out at the indicated time points, the residual lipase activities were determined by the same procedure as described above. pH-stability of the free lipase and immobilized lipase were examined in the system over a pH range between 4 and 12 for 24 h at 25 °C, respectively. The storage stabilities of free lipase, hNF-lipase, and activated hNF-lipase were determined by measuring the residual activity of the enzyme after incubation for a certain period at 25 °C. The residual activities were determined. Besides, the free lipase, hNF-lipase, and activated hNF-lipase were immersed in 50 mM sodium phosphate buffer solution (pH 7.5) at 25 °C and shaken at 200 rpm for a certain time to detect mechanical stability and stability against leaching. Then the enzyme samples were taken out at each time point, and centrifuged at 10,000 X *g* for 10 min, the residual activities in these immobilized enzymes and the supernatant liquid were measured, respectively. In addition, the reusability of hNF-lipase and activated hNF-lipase for the hydrolytic application was also evaluated. 0.5 mM 4NPA was added to 10 ml of 50 mM phosphate buffer (pH 7.5) containing 50 mg of the immobilized lipase. This reaction mixture was incubated at 37 °C for 20 min to hydrolyze 4NPA. Upon completion of one cycle, the immobilized enzyme was then separated by centrifugation. The recovered nanoflowers were washed three times with deionized water and then suspended again in a fresh reaction mixture. The residual lipase activity of each cycle was calculated by taking the enzyme activity of the first cycle as 100%.

## Additional Information

**How to cite this article**: Cui, J. *et al*. Surfactant-activated lipase hybrid nanoflowers with enhanced enzymatic performance. *Sci. Rep.* **6**, 27928; doi: 10.1038/srep27928 (2016).

## Supplementary Material

Supplementary Information

## Figures and Tables

**Table 1:** The surface characteristics of hNF-lipase and activated hNF-lipase.

Sample	S_BET_ (cm^2^/g)	Pore volume (cm^3^/g)	Pore diameter (nm)
hNF-lipase	31.04 ± 1.5	0.074 ± 0.0002	2.16 ± 0.1
activated hNF-lipase	32.42 ± 1.6	0.077 ± 0.0002	2.70 ± 0.1

**Table 2:** Quantitative estimation of the secondary structure elements of free lipase, hNF-lipase, and activated hNF-lipase.

p	α-Helix (%)	β-sheet (%)	β-Turn (%)	Random coil (%)
Free lipase	36.5 ± 1.85	29.4 ± 1.48	28.2 ± 1.41	5.9 ± 0.31
hNF-lipase	31.8 ± 1.57	34.8 ± 1.74	25.3 ± 1.27	8.1 ± 0.41
activated hNF-lipase	19.3 ± 0.97	42.5 ± 2.12	22.7 ± 1.13	15.5 ± 0.78

**Table 3:** Comparison of kinetic parameters of free lipase, hNF-lipase and activated hNF-lipase.

Enzyme	*K*_*m*_ (mM)	*V*_*max*_ (μM/mL·min)	*V*_*max*_*/K*_*m*_
Free lipase	155.34 ± 7.76	0.27 ± 0.013	0.0017 ± 0.0001
hNF-lipase	137.52 ± 6.89	0.71 ± 0.035	0.0051 ± 0.0002
activated hNF-lipase	68.05 ± 3.41	1.33 ± 0.06	0.02 ± 0.001
