# Supplementary material for: Surfactant-activated lipase hybrid nanoflowers with enhanced enzymatic performance
Source: Sci Rep. 2016 Jun 14;6:27928. doi: 10.1038/srep27928 (PMC4906385; doi:10.1038/srep27928)
Supplement: Supplementary Information [file srep27928-s1.doc]

Supplementary Information:

# Surfactant-activated lipase hybrid nanoflowers with enhanced enzymatic performance

Jiandong Cui^1, 2*^ Yamin Zhao^1^ Ronglin Liu^1^ Cheng Zhong^2^ Shiru Jia ^2*^

^1^Research Center for Fermentation Engineering of Hebei, College of Bioscience and Bioengineering, Hebei University of Science and Technology, 26 Yuxiang Street, Shijiazhang 050000, P R China

^2^Key Laboratory of Industrial Fermentation Microbiology, Ministry of Education, Tianjin University of Science and Technology, No 29, 13th, Avenue, Tianjin Economic and Technological Development Area (TEDA), Tianjin 300457, P R China

^*^ Corresponding authors:

Jiandong Cui, E-mail: [cjd007cn@163.com](mailto:cjd007cn@163.com), Tel: +86-311-81668486

Shiru Jia, E-mail: [jiashiru@tust.edu.cn,](mailto:jiashiru@tust.edu.cn,) Tel: +86-022-60601598

**Table S1. The encapsulation yield of lipase in the nanoflowers.**

| Enzyme concentration (mg/mL) | Actual concentration (mg/mL) | The supernatant concentration (mg/mL) | Encapsulation yield (%) (immobilization efficiency) |
| --- | --- | --- | --- |
| 0.025  0.1  0.25  0.5  1 | 0.02 ± 0.001  0.08 ± 0.003  0.21 ± 0.01  0.45 ± 0.02  0.92 ± 0.04 | 0.002 ± 0.0002  0.02 ± 0.001  0.064 ± 0.003  0.203 ± 0.01  0.59 ± 0.03 | 87.00%  75.00%  69.21%  54.81%  35.86% |


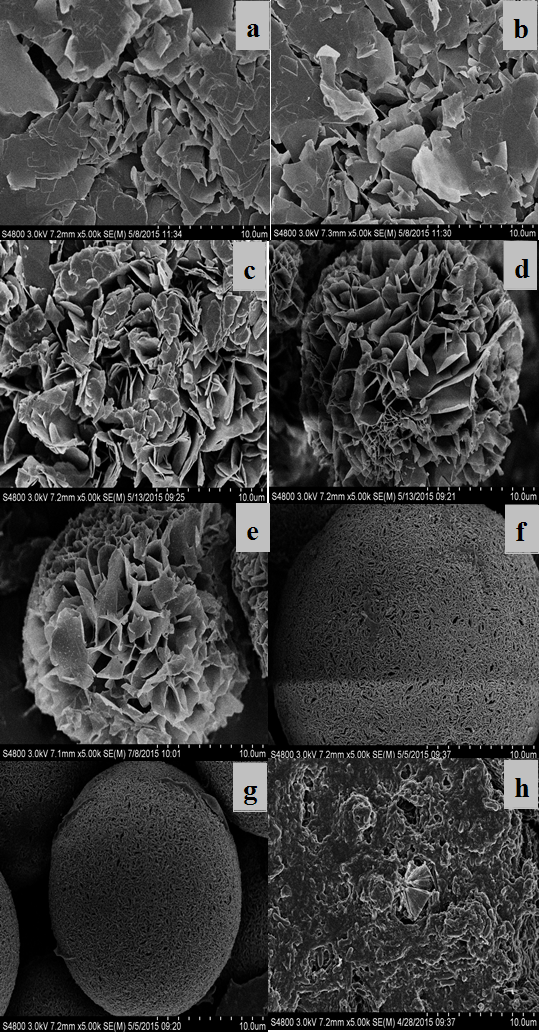


**Figure S1.** Effect of different lipase concentrations on the morphologies of nanoflowers. (a) 0.005 mg/mL, (b) 0.01 mg/mL, (c) 0.015 mg/mL, (d) 0.02 mg/mL, (e) 0.025 mg/mL, (f) 0.1 mg/mL, (g) 0.5 mg/mL, (h) 5 mg/mL.


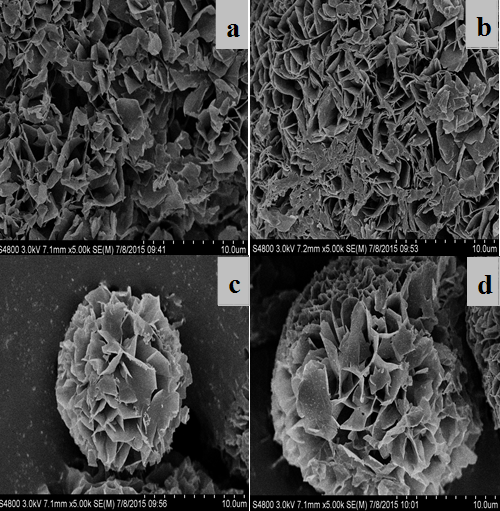

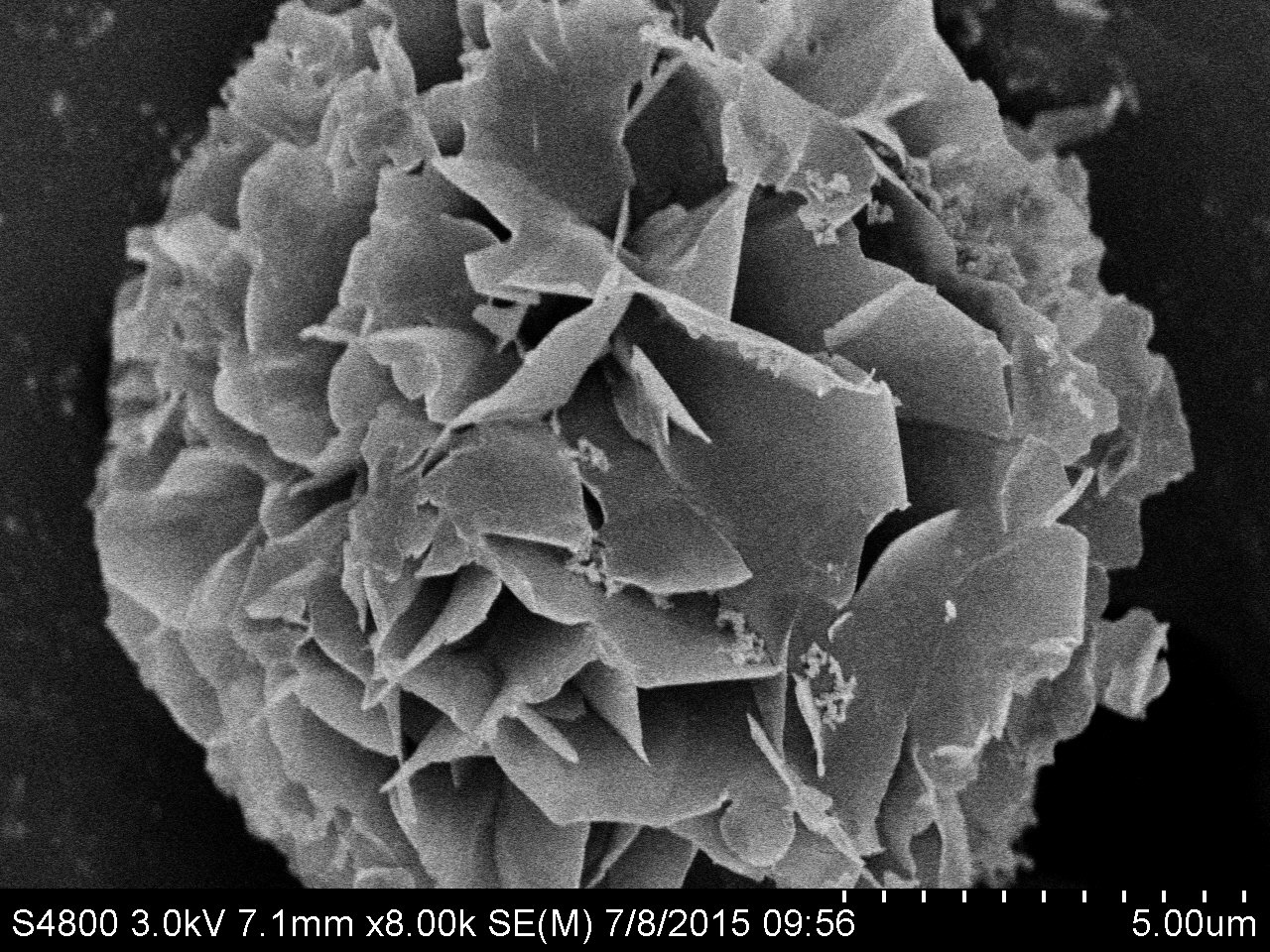


c


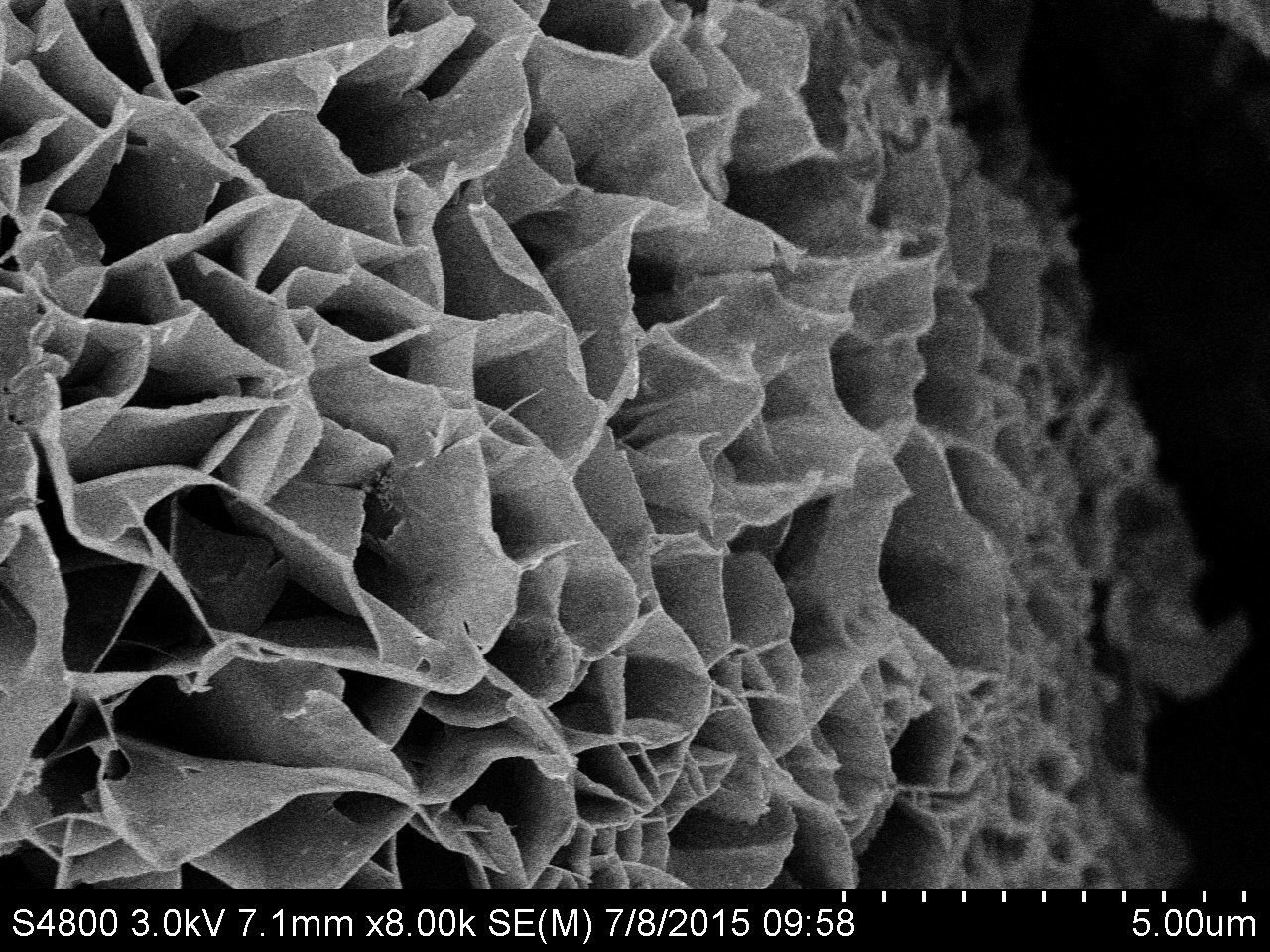


d

**Figure S2.** Effect of different CuSO_4_ concentrations on the morphologies of nanoflowers. (a) 0.4 mM, (b) 1.2 mM, (c) 2 mM, (d) 3.2 mM.


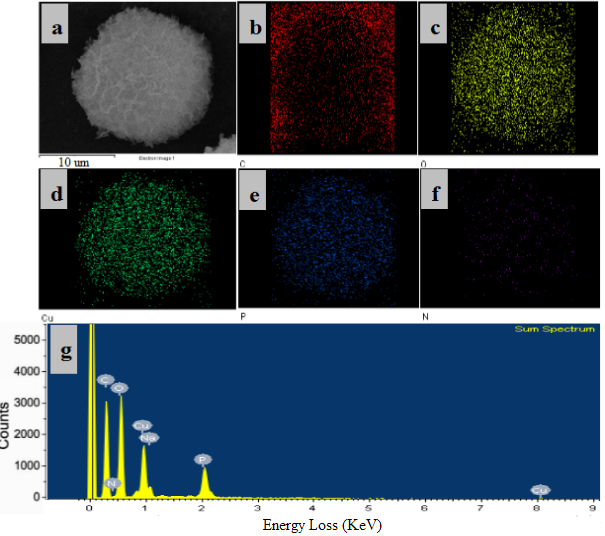


**Figure S3.** Element mapping of hNF-lipase via EDS: (a) the sample; images (b)-(f) exhibit the element sensitive maps of carbon, oxygen, copper, phosphorus, and nitrogen; (g) EDS spectrum of complete element distribution.


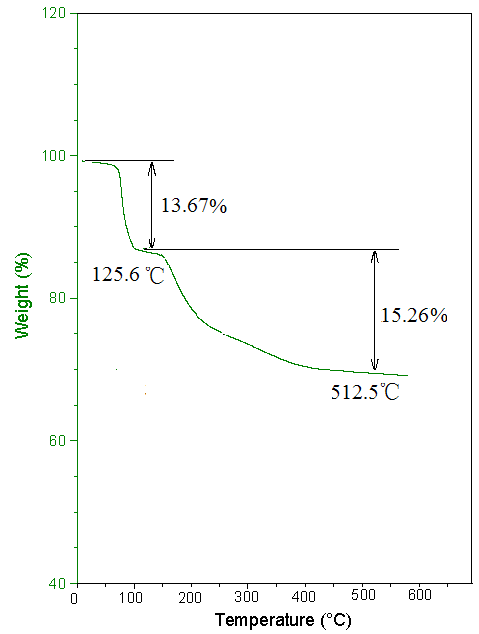


**Figure S4.** TGA curves (f) of the activated hNF-lipase


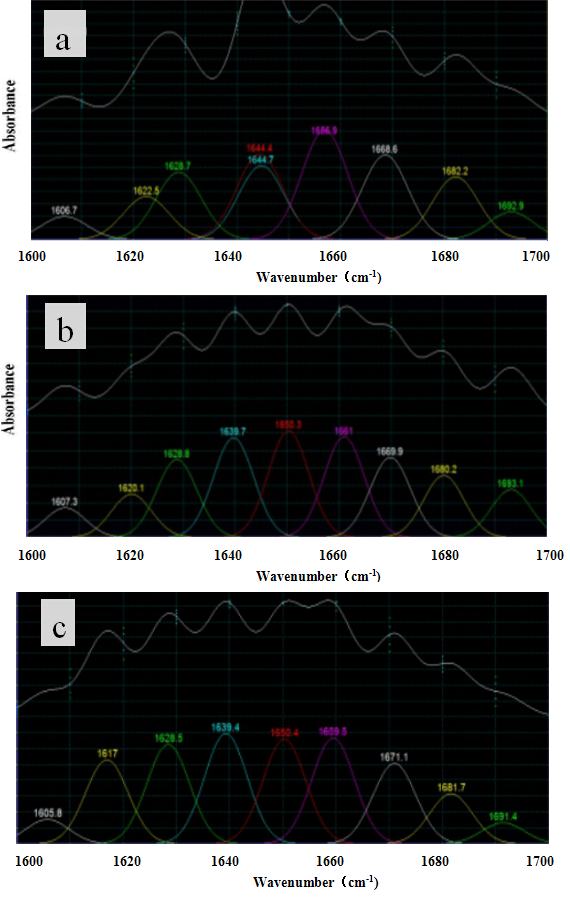


**Figure S5.** Second derivative spectrum in the region of Amide I for free lipase (a), the hNF-lipase and (b), and the activated hNF-lipase (c).


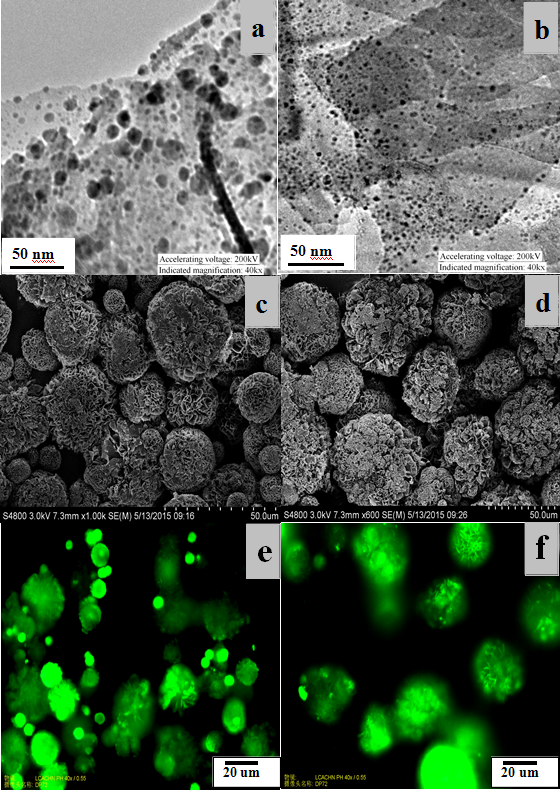


**Figure S6.** TEM images of (a) the hNF-lipase and (b) the activated hNF-lipase; SEM images of (c) the hNF-lipase and (d) the activated hNF-lipase; LCSM image of (e) the hNF-lipase and (f) the activated hNF-lipase.

(℃)

**Figure S7.** Effect of temperature on the activity of free lipase, hNF-lipase, and activated hNF-lipase.


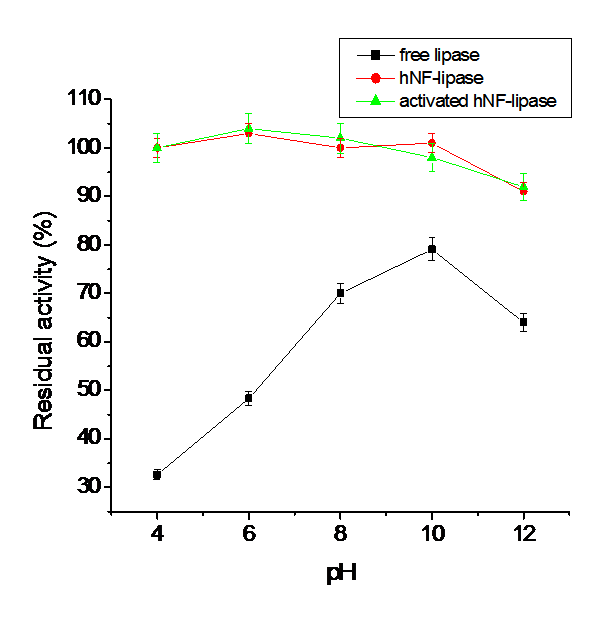


**Figure S8.** pH stability of free lipase, hNF-lipase, and activated hNF-lipase.


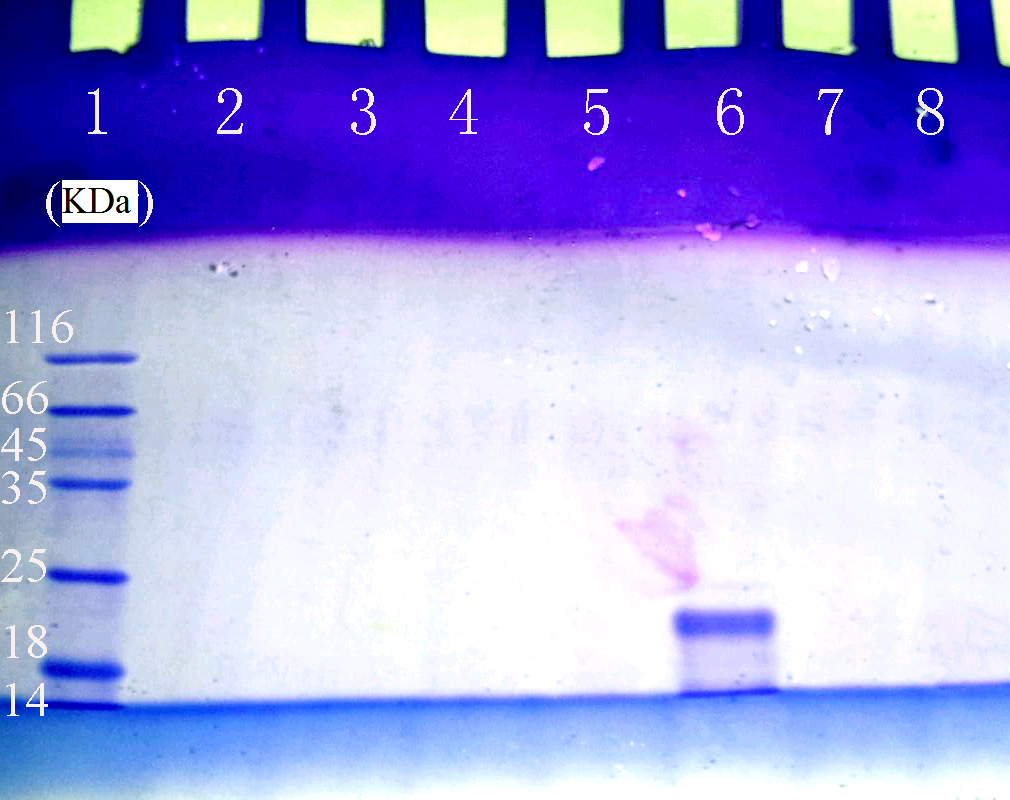


**Figure S9.** SDS-PAGE of lipase released from the activated hNF-lipase at different days of shaking. (Lane 1) Marker, (Lane 2) 5 days, (Lane 3) 10 days, (Lane 4) 15 days, (Lane 5) 20 days, (Lane 6) free lipase, (Lane 7) 25 days, (Lane 8) 30 days.


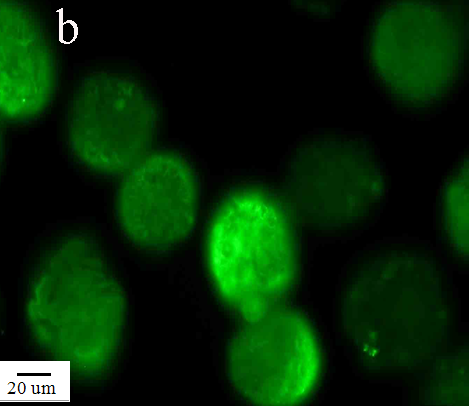

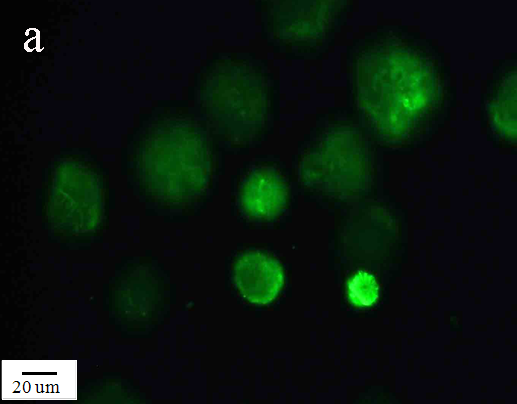


**Figure S10.** LCSM image of the hNF-lipase (a) and the activated hNF-lipase (b) after 30 days of shaking treatment.


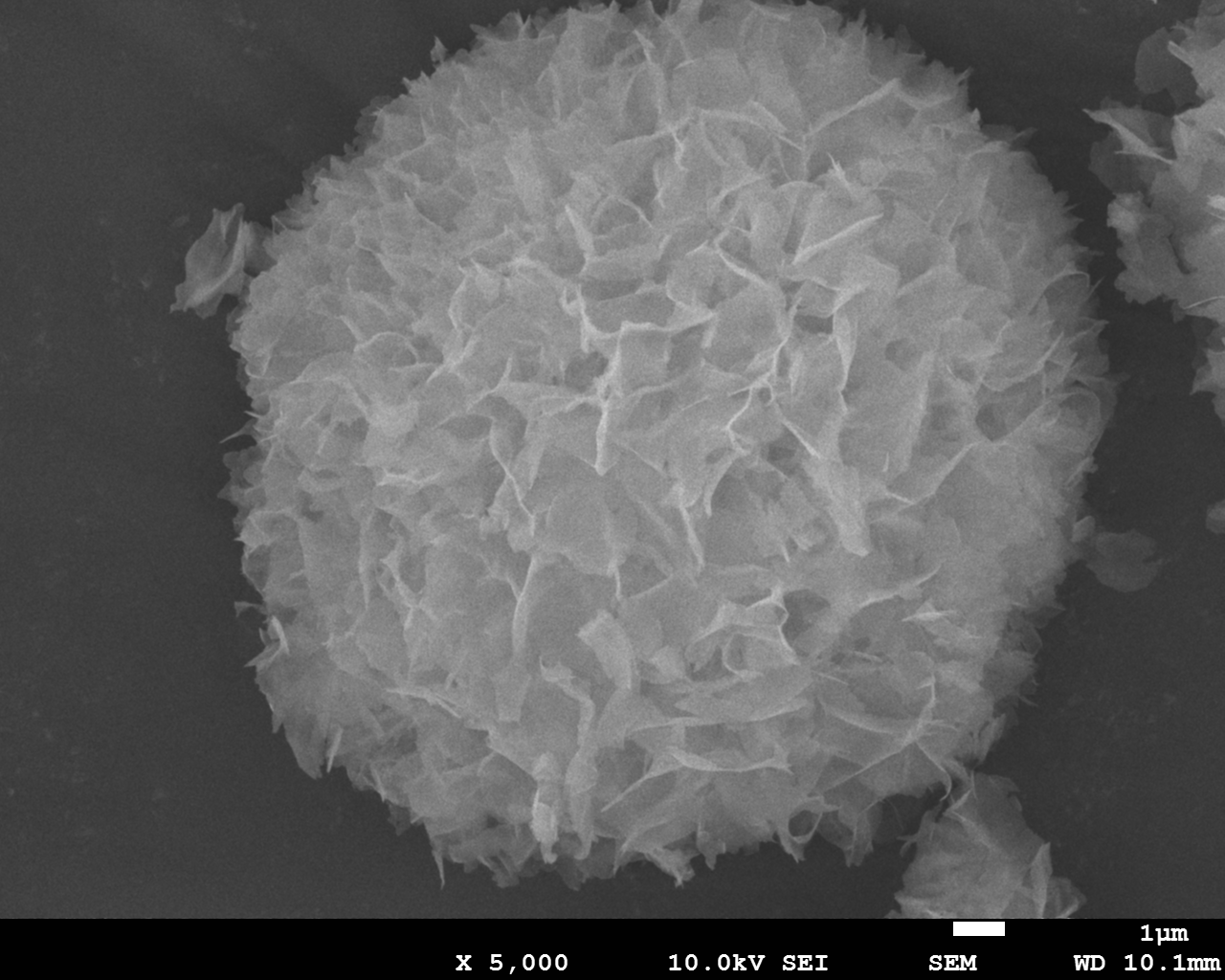


b


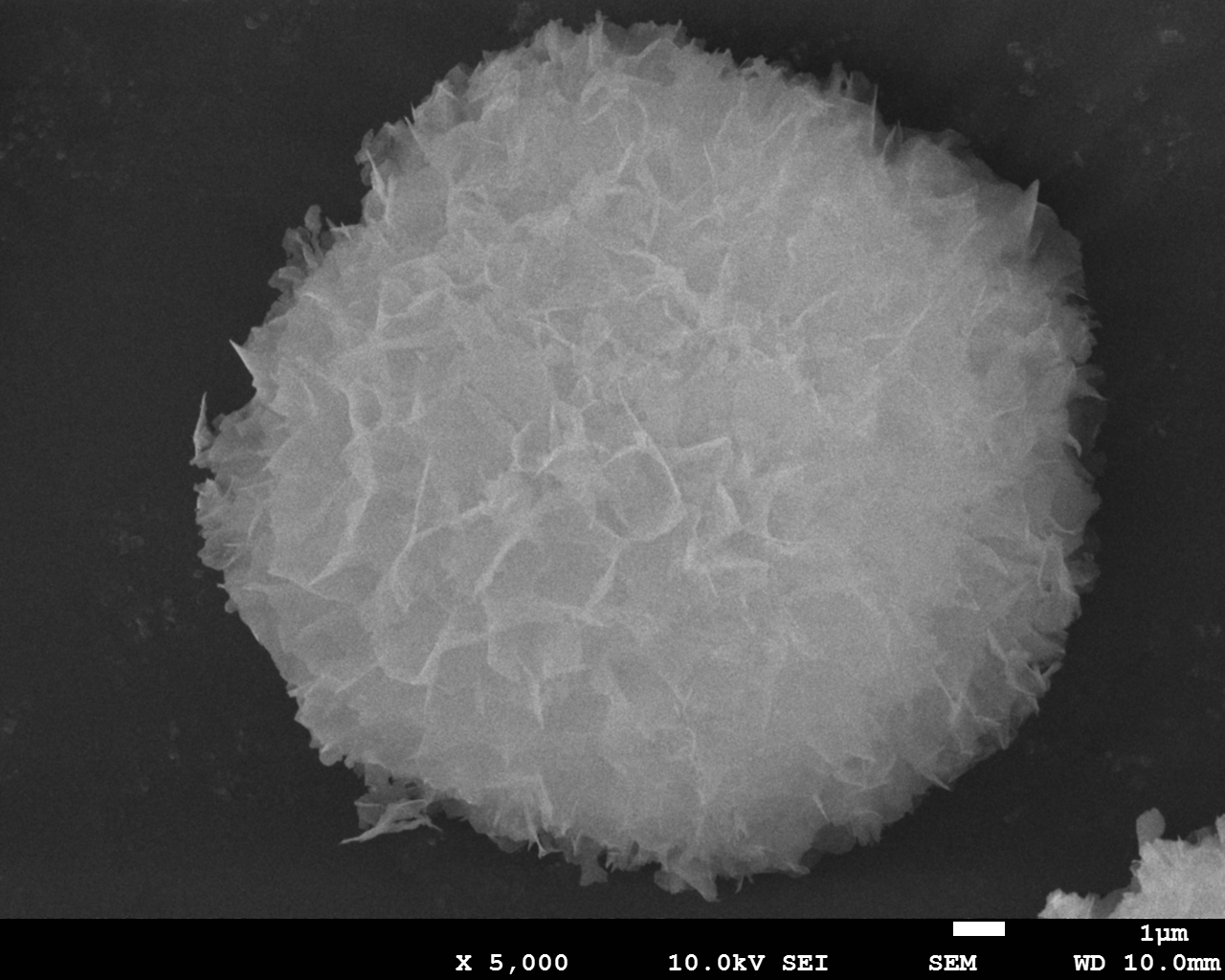


a

**Figure S11.** SEM image of the hNF-lipase (a) and the activated hNF-lipase (b) after 30 days of shaking treatment.


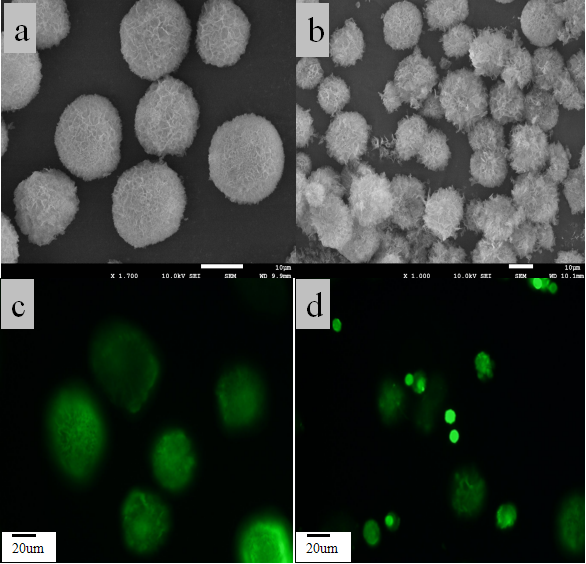


**Figure S12.** Morphology of the nanoflowers after eight rounds of successive catalytic reaction. SEM and image of the activated hNF-lipase (a) and the hNF-lipase (b); LCSM image of (c) the activated hNF-lipase and (d) the hNF-lipase;
